# Composite Material Based on Polypropylene and Modified Natural Fillers

**DOI:** 10.3390/polym16121703

**Published:** 2024-06-14

**Authors:** Ilnur Fayzullin, Aleksandr Gorbachev, Svetoslav Volfson, Yerbol Serikbayev, Abdirakym Nakyp, Nurgali Akylbekov

**Affiliations:** 1 Institute of Polymers, Kazan National Research Technological University, 68 K. Marx Str., 420015 Kazan, Russia; alexandergorbachow@gmail.com (A.G.); svolfson@kstu.ru (S.V.); abdirakym1994@mail.ru (A.N.); 2 Laboratory of Engineering Profile “Physical and Chemical Methods of Analysis”, Korkyt Ata Kyzylorda University, 29A, Aiteke bi Str., Kyzylorda 120014, Kazakhstan; nurgali_089@mail.ru

**Keywords:** polypropylene, lignocellulosic fillers, enzymatic modification, mechanical properties, high-shear forces

## Abstract

The work presents the results of a comprehensive study on obtaining compositions based on polypropylene and natural fillers modified by enzymatic preparations under high-shear forces. The experiment protocol includes determining the modification time and the ratio of water volume to the mass of natural filler (hydro modulus) during modification, which turned out to be different for each type of filler. Physical and mechanical analyses were conducted to evaluate the operational characteristics of the obtained composites, with particular attention given to comparing the modified compositions with their unmodified counterparts. The time and hydro module of the enzymatic modification of the natural fillers under consideration were investigated, which turned out to be different for each type of filler. It was found that surface modification of natural fillers improves mechanical properties; namely, the tensile strength of composites with wood and sunflower fillers increases by 10%, and the impact viscosity of composites also increases by 12% with wood and sunflower fillers. Water absorption decreases in composites, after 2 h boiling, with wood flour by 30% and with rice husk by 10%. After a 14-day test at room temperature, water absorption decreases by more than 30% in composites with rice husk. When determining the free surface energy of composites, it was found that the modification of the filler reduces the polarity of the composites in all samples, which can be interpreted as an improvement in the interaction between the filler and the polymer matrix. The findings of this research have important implications for the development of advanced polymeric materials that can be used in a wide range of applications, including automotive, aerospace, and construction industries. The results underscore the importance of surface modifications to optimize the properties of polymeric composites and provide valuable insights into the role of natural fillers in enhancing the performance of these materials.

## 1. Introduction

Currently, much attention is paid to the investigation of thermoplastic composites based on natural fillers [1,2,3]. Manufacturers of such composites increase their production volumes from year to year, as well as the range of their applications, due to their demand and declining ecological situation. Consequently, research on the utilization of agro-industrial waste from agricultural production as natural fillers becomes relevant due to their potential to reduce environmental impact and expand the opportunities for sustainable resource utilization. To obtain polymer composites filled with natural fillers with high physical characteristics, it is necessary to use mechanisms that enhance the bond between the polymer matrix and the filler. However, when obtaining polymer composites filled with natural fillers, the problem of the compatibility of the hydrophobic matrix and hydrophilic fillers arises. In this way, it is becoming important to study the modification of such polymer composites in order to improve the compatibility of components [4,5].

There are several types of modification of polymer composites with natural fillers. There are several types of modifications for polymer composites with natural fillers. One effective solution to the problem of compatibility is the introduction of special additives into the formulation, which enhance the compatibility of the components in polymer composites with natural fillers. For non-polar polymers, such as polypropylene, maleic anhydride-grafted polyolefins are widely used as a modification for compatibility [6,7,8,9,10,11]. There are also various methods of plasma surface modification for polyolefins [12,13], which, while expensive, is an environmentally friendly and versatile method. In the articles [5,14], the authors presented a review of thermo-modification as a method for improving the surface properties of polymers, especially polyolefins. This modification allows for improved wettability and adhesion properties of polyolefin surfaces, especially for composites filled with natural fillers. The work by Natalya Nosova [15] presents a method of activating the surface of polyolefins by the covalent grafting of a polyporoxide nanolayer via a free-radical mechanism. Shkuro [16] and Shpeizman [17] employ physical modification methods involving the exposure of polymer composites to various types of radiation: radio waves, UV, and streams of charged particles. The review article [18] provides a detailed description of various chemical and physical methods for the modification of lignocellulosic fillers, as well as the possibilities of combining them [19,20,21,22,23].

However, chemical and mechanical methods can generate by-products or contaminants that may be toxic or difficult to remove. This can create waste disposal issues and increase environmental impact. Additionally, there is the problem of the high cost of chemicals and equipment for modification [24].

Biological methods for the modification of polymer composites with natural fillers [25], unlike chemical and mechanical methods, can be more sustainable, efficient, and less harmful to the environment, making them attractive in many fields, including biotechnology, medicine, and ecology. There are three methods of biological modification: fungal, bacterial, and enzymatic.

The treatment of natural fillers with fungi is used to remove non-cellulosic components from the fiber surface using enzymes [26,27]. The white rot fungus from the *Schizophyllum* family produces enzymes that react with lignin components, removing it from the fiber surface and increasing its roughness [28]. This enhances the interfacial adhesion to the polymer matrix. Fungal treatment is considered an inexpensive and environmentally friendly modification method, which can make composite materials more eco-friendly.

Another method of modification involves coating the surface of natural fillers with bacterial cellulose. Bacterial cellulose is synthesized by various genera of bacteria, such as *Gluconacetobacter* (*Acetobacter*), *Agrobacterium*, *Aerobacter*, *Azotobacter*, *Rhizobium*, *Salmonella*, *Escherichia*, and *Sarcina* [29]. When added to a suitable nutrient medium in the presence of natural fibers, bacteria of these types produce bacterial cellulose, which preferably deposits onto the surface of natural fibers, creating a dense and uniform layer [30]. This bacterial cellulose coating significantly improves the physical properties of the composite material and enhances filler matrix compatibility [31].

The enzymatic modification of lignocellulosic fillers represents an innovative and environmentally-oriented approach [32]. Unlike chemical methods, enzymatic modification uses enzymes that naturally decompose without creating toxic waste. This approach significantly reduces the carbon footprint, as enzymatic modification processes typically require less energy and do not produce carbon dioxide emissions, making the method environmentally safe in the long term. Enzymatic modification is characterized by high specificity and selectivity, ensuring precise modification. This reduces the likelihood of side reactions and the formation of undesirable products. Therefore, the search for and application of biocatalysts, such as enzymes, have become key strategies in modern biotechnology. However, the disadvantages include the limited availability of enzymatic preparations and the complexity of scaling up production, as it is necessary to maintain optimal conditions (pH, temperature) for enzyme activity during storage. This underscores the need for developing methods of modification process intensification that are not only environmentally sustainable but also efficient in terms of time and resource consumption.

In this regard, let us examine in detail the mechanism of enzymatic modification. Protease enzymes belong to the group of enzymes that specialize in the hydrolysis (destruction) of proteins and have a unique structure, functioning in optimal conditions for the vital activity of organisms [33,34]. Their natural ability to operate under milder conditions becomes key in process control, which minimizes resource and energy consumption. The expansion of the specificity and range of action of enzyme preparations, such as proteases, significantly increases their attractiveness for use in various industries. In the context of the cleavage of plant fiber, proteases can be involved in the process of the dissociation of plant cellular structures. These enzymes are able to break down proteins contained in the cell walls of plants, which leads to the disruption of the wall structure and facilitates access to other cell components such as cellulose and other polysaccharides. Thus, proteases contribute to the efficient decomposition of plant materials into smaller peptides and amino acids, increasing their availability to bacteria and other microorganisms involved in the decomposition process [35].

In this process, proteins from the natural filler undergo hydrolysis, leading to their destruction. As a result, the molecular weight of the proteins decreases, and the inter-molecular interaction of the natural filler between individual chains decreases. This, in turn, improves the interaction between the polymer matrix and the natural filler, promoting a more uniform and efficient distribution of components in the composite material. The main enzymes used in this catalytic process (enzyme hydrolysis) are hydrolytic enzymes. Henriksson [36] found that enzyme treatment facilitates the disintegration of cellulose wood pulp, and George noticed [37] that treatment with enzymes enhances adhesion to the matrix and improves the physical properties of polymer composites.

For a more comprehensive understanding of the mechanism of enzymatic modification, it is necessary to analyze the structure of the natural fillers considered in the study (wood flour, rice husk, sunflower husk). The main components of natural fibers are cellulose, lignin, and hemicellulose [38,39]. Table 1 presents the classification and chemical composition of various natural fillers. As can be seen, cellulose is the main structural component of natural fillers. In addition to these three main components, plant-based materials contain non-structural components such as extracts (4–10%), inorganic ash, and water.

Cellulose is a structural component of natural fillers, and its quantity correlates with tensile strength, impact viscosity, and hardness [40]. The thermal decomposition of cellulose occurs at 200 °C, which is acceptable considering the processing temperature of polymer composite materials (180 °C) [41]. As shown in Table 1, wood flour has the highest cellulose content. This composition ratio of the filler’s chemical makeup allows more effective interaction with the polymer binder. Additionally, during high-temperature mixing, water acts as a foaming agent, hindering the formation of hydrogen bonds between the OH groups of cellulose molecules [42]. Thus, the connection between cellulose molecules weakens, leading to the complete breakdown of microfibrils (long, thin cellulose molecules intertwined with each other) into individual linear molecules [21]. As a result, a porous material with reduced adhesion between the polymer matrix and natural filler is obtained, exhibiting low strength.

Lignin is a complex polymer compound containing polar groups (-OH). The degradation of lignin at 180 °C results in the release of carbon dioxide during mixing [43], reducing the density and strength of the composite [44]. The introduction of polar groups of lignin into the composition of non-polar polypropylene macromolecules increases viscosity because it disrupts the regularity of the polymer chain structure. Additionally, lignin is a photosensitive material, and its degradation under UV light causes the discoloration of wood and wood–polymer composite materials. Under the influence of UV light, lignin changes in color from brownish to gray.

In addition to cellulose and lignin, the third main component is hemicellulose, which forms amorphous three-dimensional structures surrounding cellulose fibers. Hemicelluloses include xylans, arabinoxylans, glucuronoxylans, glucomannans, galactomannans, and xyloglucans [45]. Like lignin, hemicellulose undergoes degradation at temperatures of 160–180 °C. At high mixing temperatures, it forms reactive acetic acid, leading to gradual corrosion of the mixing equipment.

Silica in rice husk forms hydrophilic silicate–galactose complexes, which hinder moisture penetration [46]. Composite materials made from rice husk, which contain up to 19% silica [47], exhibit lower moisture absorption compared to wood flour and sunflower husk, which contain less than 1% silica.

The study hypothesizes that the enzymatic modification of natural filler under high-shear deformation can enhance the performance properties of polypropylene-based composite materials. The research aims to determine the optimal technological parameters of such modification and establish the physico-mechanical characteristics of the obtained composites. The scientific novelty of the work lies in identifying the mechanism of the enzymatic modification process under high-shear forces of natural fillers by disrupting the internal bonds of filler particles, leading to changes in their morphology and improvement in the performance characteristics of polymer composite materials.

## 2. Materials and Methods

### 2.1. Materials

As the polymer binder, extrusion-grade polypropylene 4215M with a melt flow index of 7–10 g/10 min and a density of 900 kg/m^3^ produced by PJSC SIBUR Holding (Nizhnekamsk, Russia) was used at a dosage of 50% by weight, as this dosage is optimal for the production of polymer composites with natural fillers [48,49]. To enhance the thermal stability of the polymers during processing, an Irganox 1010 brand (Kazan, Russia) antioxidant was used. Wood flour produced by LLC PF “Lignum-Resource” (Zelenodolsk, Russia) with a particle size of 0.18 mm, sunflower husk, and rice husk produced by LLC “Agrodar” (Krasnodar, Russia) with an average particle size ranging from 0.1 to 1 mm were used as fillers.

Enzyme preparations under conditions of high-shear forces increase the reactivity between the components of natural fillers in a humid environment, namely, the protein, starch and lipids contained therein. Under these conditions, lipids are hydrolyzed to triglycerides, starch to amylose, like substances and proteins, and partially to oligomeric compounds.

To select the modifier for natural fillers, the content of protease in various complexes of enzyme complex was analyzed. Protease is a hydrolase class enzyme which breaks peptide bonds -CO-NH- between amino acids in proteins, converting them into polypeptides and single amino acids [50]. Therefore, complexes of enzyme preparations with different protease content of the following brands were considered objects of research: Agroxyl and Agrocell manufactured by Agroferment LLC (Staroseslavino, Russia), Allzyme Vegpro manufactured by Alltech (Lexington, MA, USA). As a result of preliminary experiments, a complex of enzyme preparations of the Allzyme Vegpro brand with the highest protease activity (at least 7500 μ/g) was selected for modification. Composition formulations are presented in Table 2.

### 2.2. Preparation or Modification of the Composition

The process of obtaining polymer composites was carried out in several stages. In the first stage, natural fillers were modified by enzymes under conditions of high-shear deformation on a two-rotor closed-type mixer, “Measuring Mixer 350E”, manufactured by Krauss Maffei (Munich, Germany). At high shear deformation, intense abrasion of the natural filler occurs between the walls and rotors in the presence of the enzymatic preparation. This significantly accelerates the modification process by increasing the available surface area of the natural filler for interaction with the modifier.

For enzymatic modification, it was necessary to determine the technological parameters of mixing various fillers. These parameters include the degree of mixer loading, the hydraulic module, and the time interval for heating the mixture to a critical temperature of 70 °C. It is important to note that this critical threshold is due to the need to prevent the destruction of enzymes [51,52]. The degree of loading of the mixer directly affects the torque and mixing temperature, while a higher degree of loading corresponds to a higher torque and an increase in mixing temperature. On the other hand, the hydraulic module plays a role in reducing torque and reducing the mixing temperature. These parameters are key in determining the conditions for the modification process. To determine the technological parameters, the kinetics of the torque change, as well as the mixing temperature of the material during enzymatic modification were recorded on the computer of the mixing machine.

The second stage of the work consisted of obtaining composite materials based on polypropylene and modified natural filler on a closed-type two-rotor mixer, as mentioned earlier in the first stage. The mixing temperature was 180 °C; the mixing time was 11 min.

At the third stage of the work, samples were prepared for testing. The injection molding machine Glassix CX 50-180 manufactured by Krauss Maffei (Germany) was used. The injection pressure was 1100 bar, the temperature in the zones was T_0_ = 50 °C, T_1_ = 180 °C, T_2_ = 185 °C, T_3_ = 190 °C, T_4_ = 195 °C, T_5_ = 200 °C, where T_0_ is the raw material loading zone, and T_5_ is the extruder head.

### 2.3. Measurements

#### 2.3.1. Determination of Physical and Mechanical Properties

Mechanical tests to determine deformation and strength characteristics were conducted on a UGT-AI7000-M Gotech machine (Taipei city, Taiwan) according to ISO 527-2:2012 standard at a temperature of 23 ± 2 °C and a deformation speed of 5 mm/min. Hardness was measured using a TVR-ATS durometer, East-7 (Russia), with an OS-2 Hildebrand operational stand (Germany) according to ISO 868 standard (Shore D scale). The test result is the arithmetic mean of three parameters.

#### 2.3.2. Determination of Impact Strength

The determination of impact strength by the Charpy method was carried out on a GT-7045-MDL Gotech machine (Taiwan) with a pendulum energy of 5.5 J, impact speed of 3.46 m/s, and a temperature of 23 ± 2 °C according to ISO 179-1:2010 standard. The test result is the arithmetic mean of three parameters.

#### 2.3.3. Determination of Melt Flow Index

The melt flow index was determined by Method A (by mass) on a UGT-7100-MIBH Gotech apparatus (Taipei city, Taiwan) at a temperature of 190 °C, a load of 5 kg, and preheating of the material for 5 min according to ISO 1133-1:2011 standard. The diameter of the die hole was 2 mm. The test result is the arithmetic mean of three parameters.

#### 2.3.4. Determination of Water Absorption

Water absorption was evaluated over 14 days at 23 °C and after immersion in boiling water for 2 h according to ISO 62. The mass fraction of absorbed water for each specimen was calculated using the formula:

(1) $$\alpha =\frac{m_{2}-m_{1}}{m_{1}}\times 100\%,$$

*m*_2_ is the mass of the specimen after soaking in water, gram (g).
*m*_1_ is the mass of the specimen after initial drying and before immersion into water, gram (g).

The arithmetic average of three indicators obtained at the same duration of exposure to water is taken as the test result.

#### 2.3.5. Optical (Light) Microscopy

Optical microscopy was performed using a laboratory polarizing microscope Axioskop 40 Pol Zeiss (Germany), designed for observations in transmitted and reflected light at 100× magnification. This method was used to study the surface structure of composites with modified and unmodified filler and to determine the distribution of natural filler in the polymer composite. The method involved observing the light field in reflected and polarized rays of transmitted light. The specimen was illuminated with polarized light. The filled polymer composite partly absorbed and partly scattered the incident light, resulting in the images observed.

#### 2.3.6. Determination of Free Surface Energy

The study of the effect of enzymatic modification on the surface characteristics of a composite material was carried out using samples made by casting on an injection molding machine.

The evaluation of the free surface energy ${(\gamma }_{s})$ of the polymer composite and its acid–base ($\gamma_{s}^{ab}$) and dispersion ($\gamma_{s}^{d}$) components was carried out by measuring the contact angles of wetting the surface of the samples with test liquids. Methods based on the Owens–Wendt Equation (2) were used to analyze the obtained data [53].

(2) $$0.5\gamma_{1}\left(1+cos\;\theta \right)={\left(\gamma_{l}^{d}\right)}^{0.5}{\left(\gamma_{s}^{d}\right)}^{0.5}+{\left(\gamma_{l}^{ab}\right)}^{0.5}{\left(\gamma_{s}^{ab}\right)}^{0.5}$$

The dependence can be represented as a straight line in Fawkes coordinates (3).

(3) $$x={\left(\gamma_{l}^{ad}/\gamma_{l}^{d}\right)}^{0.5},\;y=0.5\gamma_{l}\left(1+cos\;\theta \right)/{\left(\gamma_{l}^{d}\right)}^{0.5}$$

According to the concept of Fawkes [54], free surface energy can be decomposed into the sum of components due to various interaction forces. In the context of this concept, it is sufficient to take into account only two main components: acid–base and dispersion (4). The error of this method is estimated at no more than 2%.

(4) $$\gamma_{s}=\gamma_{s}^{ab}+\gamma_{s}^{d}$$

**Figure 1:**
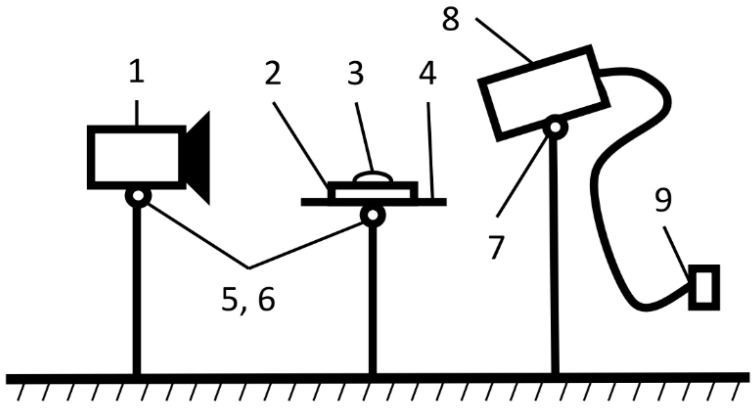
Installation diagram for determining contact wetting angles: 1—cathetometer; 2—composite material to be tested; 3—liquids; 4—table holder; 5, 6, 7—adjusting screws; 8—illuminator; 9—power supply.

To measure the contact angle of wetting, a special stand was used, shown in Figure 1. The method used was based on the principle of a sitting drop in a cell with a hydraulic shutter and included the use of a KM-8 cathetometer manufactured in the USSR (Izyum Instrument-Making Plant, USSR, Kharkiv), equipped with a micrometer nozzle. The measurement process was carried out as follows: the tested composite material (2) was placed on a special table holder (4), after which the lighting lamp (8) was switched on. Then, the following steps were performed: the screw (5) was unscrewed, and the cathetometer (1) was adjusted at the same level as the test sample. Then, using a micro-syringe, a drop (3) of liquids with a volume of 1 m^3^ was applied to the surface of the test sample at its edge facing the cathetometer (1).

To ensure the accuracy and reproducibility of the results, each of the experiments involved applying at least 5 drops of liquid of the same size using a micro-syringe. The diameter of each drop was 2–3 mm, providing standardized measurement conditions. The temperature in the room where the measurements were carried out was controlled and maintained at 20 ± 1 °C. The surfaces of the polymer composite samples were previously degreased with acetone to eliminate the influence of contamination on the results. The relative measurement error was 1.9%. The contact angles of wetting were measured 2 min after application of the drop, taking into account the ability of the polymer composition to absorb water on its surface.

## 3. Results of the Discussion

Based on the analysis of the influence of various moisture ratios on the change in torque during modification, the optimal ratio of water volume to the mass of natural filler (hydration modulus) was determined. Insufficient or excessive water content resulted in a decrease in torque, negatively affecting the efficiency of modification. During the fermentative modification of wood flour, it was found that the hydration modulus was 1:2. Similar ratios were revealed for mixing sunflower husk with a modifier (3:5) and a modifier with rice husk (1:2).

**Figure 2:**
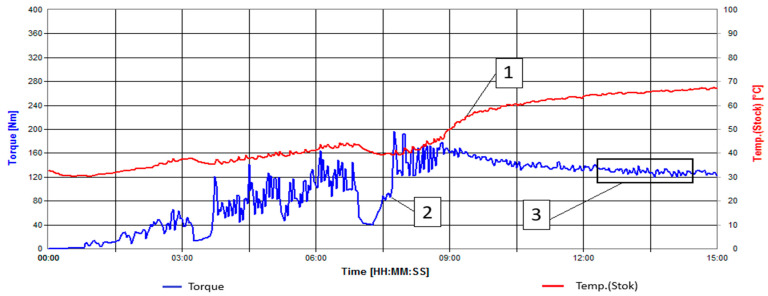
Kinetic curves of the enzyme modification process of wood flour. 1—Temperature [°C], 2—Torque [N*m], 3—Region where the torque curve reaches a plateau.

To determine the modification time of wood flour using kinetic curves (Figure 2), a modification time of 13 min was revealed. This duration was chosen because, at this time interval, the torque reaches a plateau, and the temperature does not reach the critical value of 70°.

**Figure 3:**
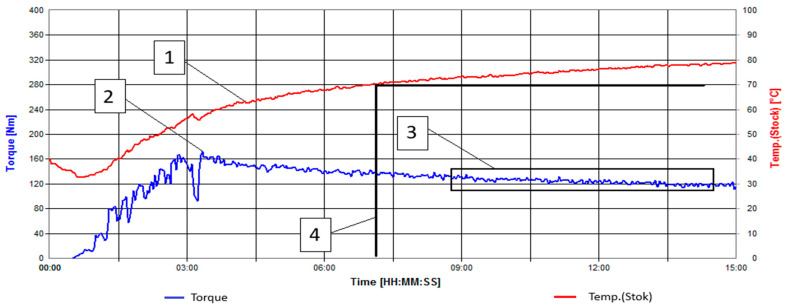
Kinetic curves of the enzyme modification process of sunflower husk. 1—Temperature [°C], 2—Torque [N*m], 3—Region where the torque curve reaches a plateau, 4—Attainment of the critical modification temperature.

The modification time of the sunflower husk was less than 7 min (Figure 3), as the temperature reached the critical value at this time interval.

**Figure 4:**
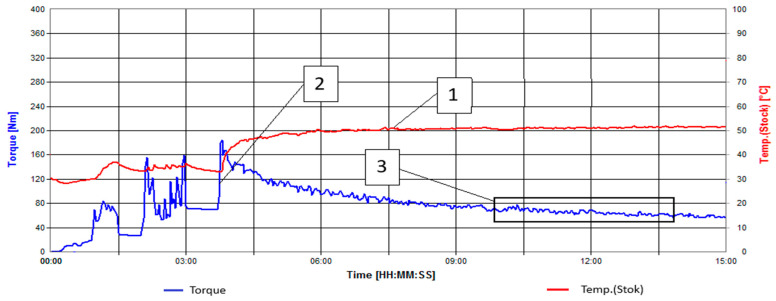
Kinetic curves of the enzyme modification process of rice husk. 1—Temperature [°C], 2—Torque [N*m], 3—Region where the torque curve reaches a plateau.

During the enzyme modification of rice husk (Figure 4), the modification time is 11 min, as the torque reaches a plateau at this time interval, and the temperature does as well.

The next step involved obtaining composite materials based on polypropylene and modified natural filler, followed by conducting physical tests for the compositions obtained according to the optimal modification parameters.

**Figure 5:**
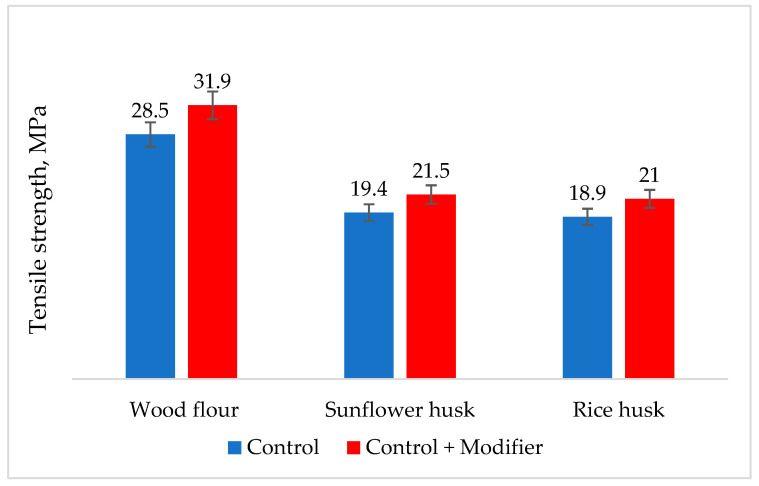
Effect of composite modification on tensile strength.

It was found that samples with modified filler are superior to the control sample, which contains filler untreated with the enzyme complex. Data from Figure 5 show that modification results in tensile strength increased by 10% in all samples. Due to the presence of the enzyme modifier in the natural filler, proteins undergo hydrolysis, resulting in a decrease in molecular weight and a reduction in intermolecular interaction of the natural filler between individual chains, thereby improving the interaction between the polymer matrix and the natural filler [55,56].

**Figure 6:**
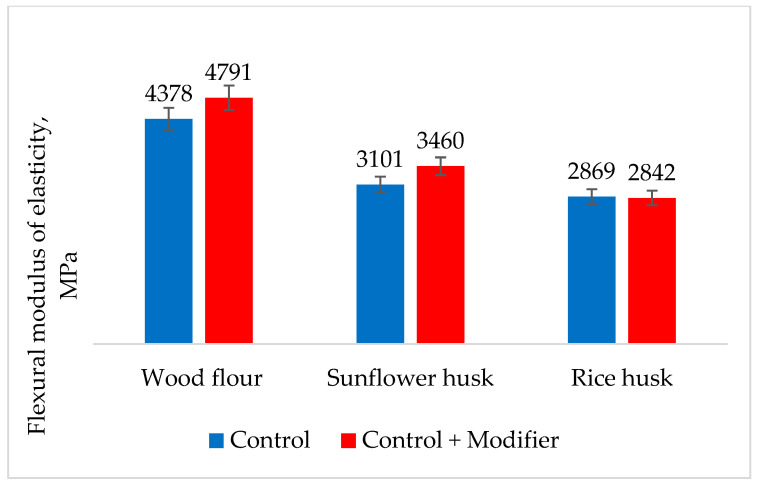
The effect of modification of composites on the modulus of elasticity during bending.

The modulus of elasticity at bending for modified composites with wood flour and sunflower husk increases by 10% compared to the control sample (Figure 6). For the composition with rice husk, the modulus of elasticity at bending changed insignificantly, most likely due to the low content of cellulose and hemicellulose, which underwent less modification, as well as due to the high content of silica in the filler.

**Figure 7:**
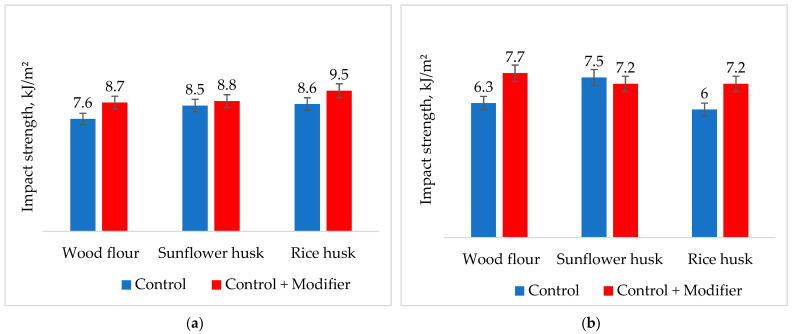
Effect of composite modification on the Charpy impact strength at +23 °C (**a**) and −40 °C (**b**).

From the data in Figure 7, it is clear that the Charpy impact strength of samples with enzyme modification increases both at lowered and room temperatures. Specifically, for compositions with modified wood flour at +23 °C, a 15% increase in impact strength is observed, and with rice husk, it increases by 10%. At −40 °C, the impact strength of the compositions increased by 20% in both cases. This increase in impact strength is likely due to the high cellulose content in wood flour and rice husk. Modification of sunflower husk has little to no effect on the impact strength at both temperatures.

**Figure 8:**
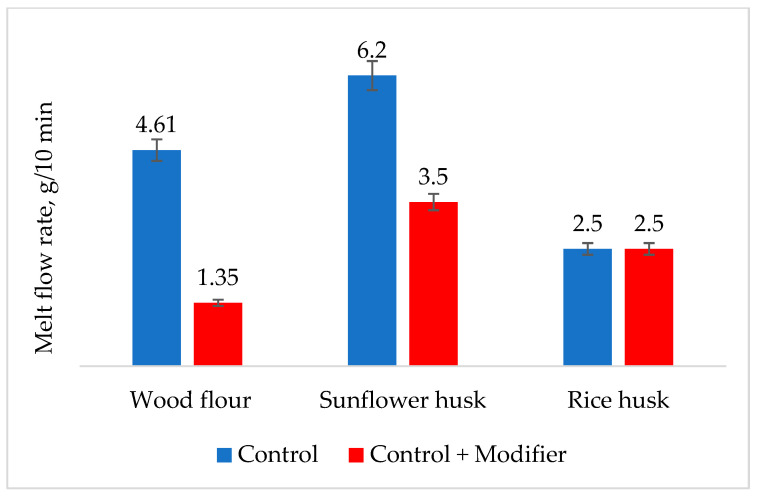
Effect of composite modification on melt fluidity at 190 °C and 5 kg load.

The melt flow index of composites is crucial as a marker for selecting the optimal processing conditions for polymer-filled composites on an industrial scale. As evident from Figure 8, during the modification of natural fillers, the melt flow index of compositions with wood flour and sunflower husk decreases by 70% and 40%, respectively, while it remains unchanged for rice husk. The decrease in melt flow index is likely associated with an increase in the dispersity of the natural filler during enzyme modification.

**Figure 9:**
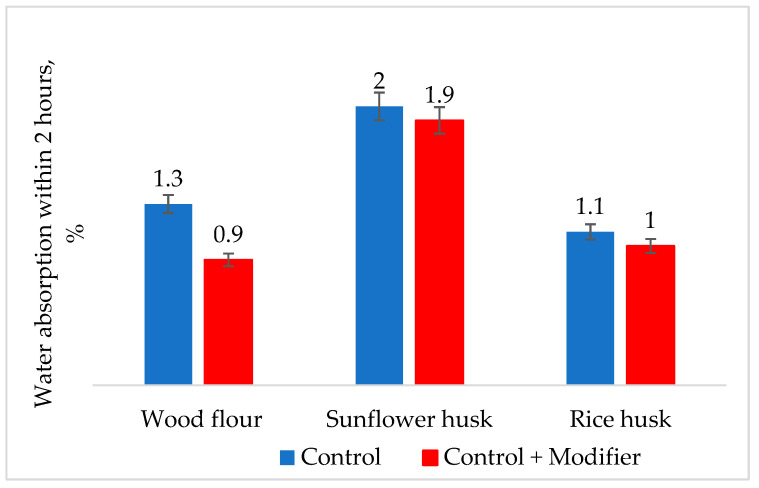
Effect of composite modification on water absorption for 2 h during boiling.

When investigating the dependency of water absorption during a 2 h boiling test (Figure 9), it is evident that for samples with modified wood flour, water absorption decreased by 30%. For samples with rice husk, this parameter decreased by 10%, while for samples with modified sunflower husk, water absorption remained unchanged compared to the control sample.

**Figure 10:**
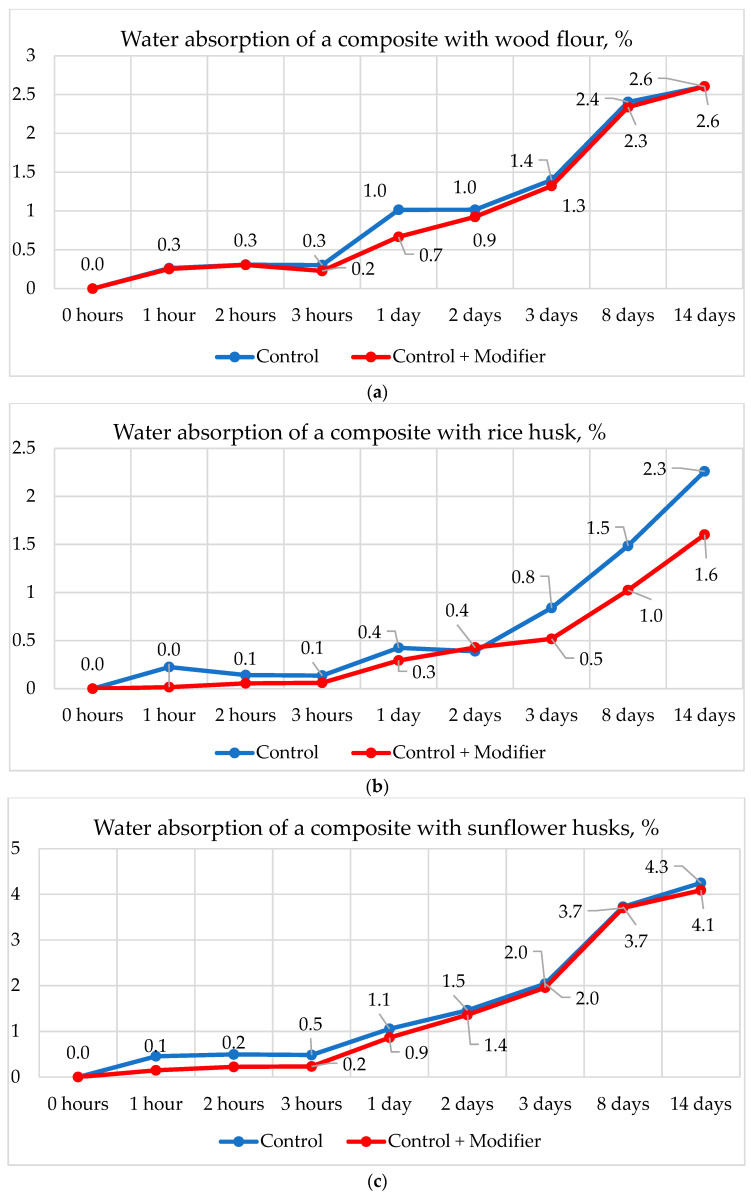
Effect of composite modification on water absorption for 14 days at 23℃. (**a**)—with wood flour, (**b**)—with rice husks, (**c**)—with sunflower husks.

When studied at 23 °C over 14 days, the water absorption of the composite with wood flour and sunflower husk (Figure 10a,c) changed insignificantly. The most significant changes were observed in the composite with rice husk (Figure 10b), particularly on the 2nd day of testing, where a reduction in water absorption by 37% was detected, on the 8th day by 33%, and on the 14th day by 30%.

**Figure 11:**
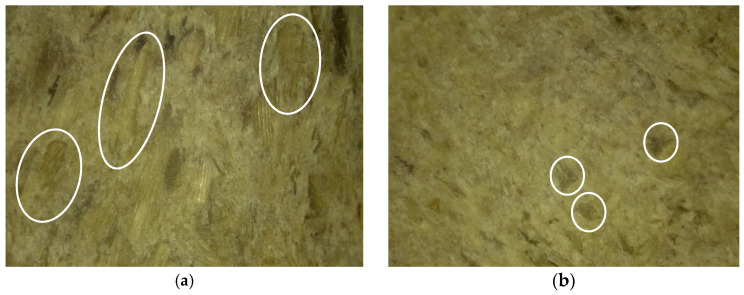
Microstructure of the surface of the polymer composite filled with wood flour. (**a**)—Control, (**b**)—Control + Modifier.

**Figure 12:**
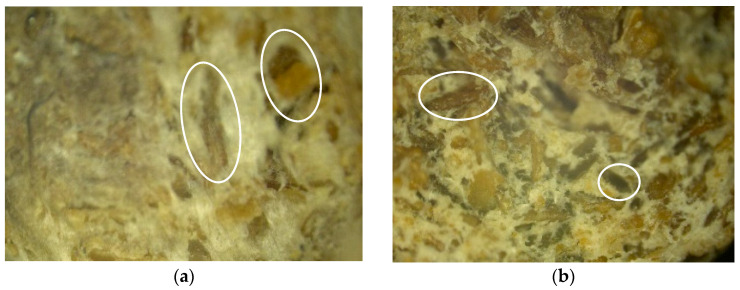
Microstructure of the surface of a polymer composite filled with rice husks. (**a**)—Control, (**b**)—Control + Modifier.

**Figure 13:**
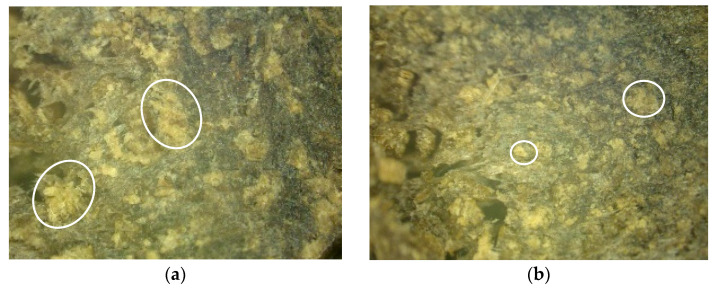
Microstructure of the surface of a polymer composite filled with sunflower husks. (**a**)—Control, (**b**)—Control + Modifier.

Optical microscopy was conducted to study the microstructure of the surface of composites with modified and unmodified fillers and to determine the distribution of natural filler in the polymer matrix. Figure 11, Figure 12 and Figure 13 show images of the composites at 100× magnification.

As seen in Figure 11, the control sample (a) contains needle-like particles, whereas the modified sample (b) exhibits plate-like particles of significantly smaller size.

In Figure 12, the control sample (a) contains both needle-like and plate-like particles, whereas the modified sample exhibits smaller plate-like particles.

In Figure 13, it is evident that the sizes of sunflower husk particles decreased after modification.

During the microstructural analysis of the surface of composite materials, the absence of macro- and micro-defects such as cracks and pores was observed in materials using both the control filler and those using the modified filler. Additionally, a uniform distribution of the filler within the polymer matrix was observed without the formation of agglomerates or other heterogeneities. This phenomenon indicates a high degree of dispersion of components during mixing, suggesting efficient interaction between phases and providing homogeneous mechanical and physicochemical properties of the material.

**Figure 14:**
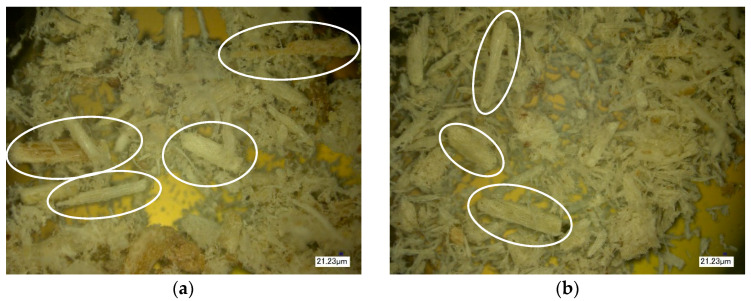
Microstructure of the surface of wood flour. (**a**)—Control, (**b**)—Control + Modifier.

**Figure 15:**
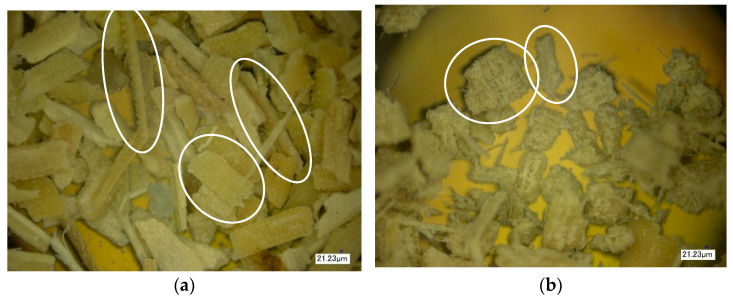
Microstructure of rice husk surface. (**a**)—Control, (**b**)—Control + Modifier.

**Figure 16:**
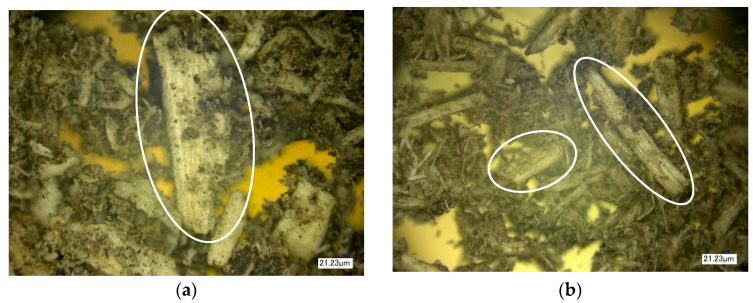
Microstructure of sunflower husk surface. (**a**)—Control, (**b**)—Control + Modifier.

The next stage of the study involved the analysis of the surface structure of unmodified (control) and modified natural fillers using optical microscopy. Figure 14, Figure 15 and Figure 16 show images of the fillers at 100× magnification.

During the analysis of the surface microstructure, it was found that enzymatic modification significantly affects the physical characteristics of natural filler particles.

In particular, the modification of wood flour leads to significant morphological destruction, accompanied by a reduction in the size of needle-like particles and an increase in the fibrous structure of the natural filler (Figure 14). When modifying rice husk, it is observed that the particles undergo slight changes in integrity, but there is surface swelling of the particles and an increase in the content of microfibers on their surface, which increases the surface area of the filler (Figure 15). Similar changes are also observed in the modification of sunflower husk, which leads to particle destruction and an increase in the fibrous structure (Figure 16), similar to what is observed with wood flour. This indicates the similarity in morphology and the influence of modification processes on wood flour and sunflower husk particles.

From the figures, it is evident that enzymatic modification under high-shear forces contributes to the disruption of internal bonds in the structure of natural filler particles, leading to the observed changes in their morphology. The increase in the surface area of filler particles contributes to the enhancement of the physical and mechanical properties of the polymer composite material.

The results of calculating the free surface energy (FSE) and its components according to the data of wetting the control and modifiable samples with test liquids using graphical Fawkes dependencies are shown in Table 3. The acid–base component ($\gamma_{s}^{ab}$), the dispersion component ($\gamma_{s}^{d}$) and the total ($\gamma_{s}$) were determined in the work FSE of pure polypropylene, as well as filled with non-modified (Control) and modified (Control + Modifier) natural fillers.

**Figure 17:**
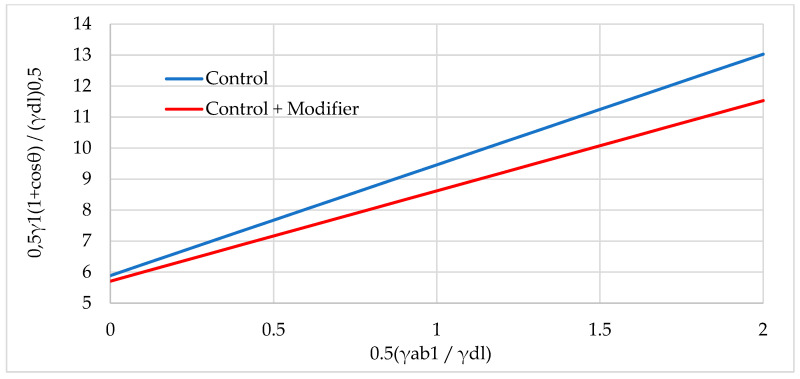
Graphical dependence in Fawkes coordinates for calculating the total FSE of samples with wood flour: not modified (Control) and modified (Control + Modifier).

**Figure 18:**
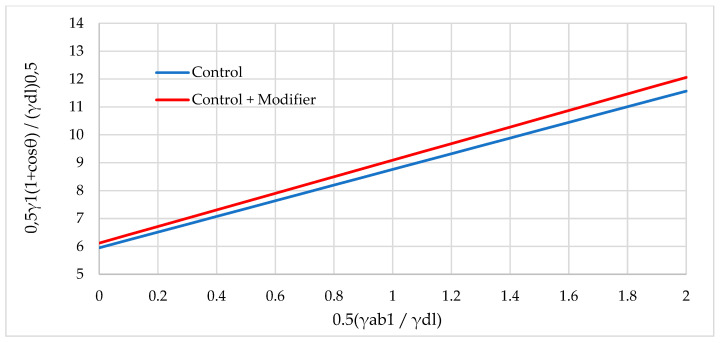
Graphical dependence in Fawkes coordinates for calculating the total FSE of samples with rice husk: not modified (Control) and modified (Control + Modifier).

**Figure 19:**
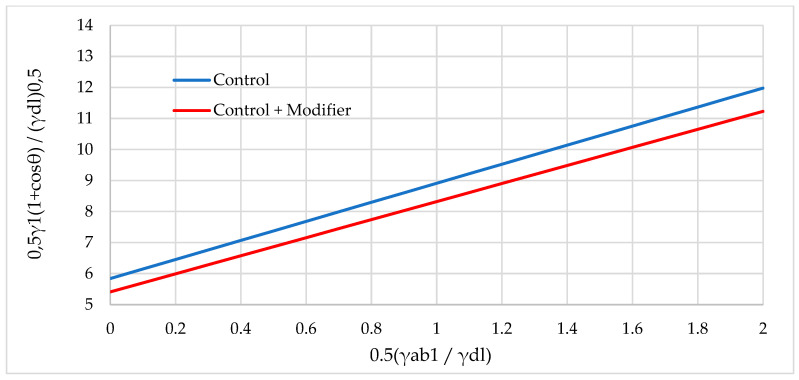
Graphical dependence in Fawkes coordinates for calculating the total FSE of samples with sunflower husks: not modified (Control) and modified (Control + Modifier).

Figure 17, Figure 18 and Figure 19 show graphical dependencies in Fawkes coordinates for calculating the total FSE.

The results show that the introduction of a vegetable filler leads to a significant increase in the polarity of the sample surface. This indicates that the polar filler is present in the surface layer of the polymer composite. At the same time, the enzymatic modification of the filler reduces the polar component of the FSE. Obviously, the modification improves the interaction of the filler with the polymer matrix and a more uniform distribution in the absence of agglomerates.

## 4. Conclusions

The conducted research allowed determining the technological parameters of enzyme modification of natural fillers under conditions of high shear deformation. It was found that the modification time for wood flour is 13 min with a hydration modulus of 1:2; for sunflower husk, it is 7 min with a hydration modulus of 3:5; and for rice husk modification, it is 11 min with a hydration modulus of 1:2.

During the investigation of the physical properties of polypropylene compositions with modified wood flour, rice husk, and sunflower husk, it was found that tensile strength and the modulus of elasticity increase in all compositions. Impact toughness at 23 °C and −40 °C increases only in compositions with wood flour and rice husk. The melt flow index remains unchanged in compositions with rice husk but significantly decreases in compositions with wood flour and sunflower husk. Water absorption after two hours of boiling decreases in all compositions, while at room temperature over 14 days, it decreases only in the composition with rice husk.

Upon analysis of the surface structure of the composite materials, the absence of defects and the uniform distribution of the filler in the polymer matrix was observed, indicating high-quality blending of the components. It was also noted that enzymatic modification of the filler leads to a reduction in the polar component of the composite surface, which can be interpreted as an enhancement of the interaction between the filler and the polymer matrix.

From the analysis of the presented results, it becomes obvious that, in some cases, the modification process has a negligible effect on the parameters of the resulting composites. However, it is necessary to carefully consider this statement since the significance of the study is manifested in its connection with other fields and possible applications. For example, minor parameter changes can make a significant difference in industrial areas. At the same time, the research process itself helps to better understand the mechanisms of interaction between the components of the material. This becomes the basis for further research and innovation in the field of materials science and composite technology.

The findings of this study contribute to the development of materials with enhanced characteristics for various industrial sectors, such as construction materials, automotive manufacturing, and packaging, thus reducing energy and resource consumption in composite material production and improving the environmental sustainability of industrial operations. The application of the enzymatic modification of fillers not only stimulates the development of more efficient methods for processing natural waste and utilizing them in manufacturing processes but also opens up opportunities for further research. Future studies will focus on the stability of composites to ultraviolet radiation, chemical agents, and biological corrosion. The water absorption of composites will be investigated under different operational conditions, including exposure to seawater and aggressive environments, as well as the possibility of using mixed lignocellulosic fillers to enhance the characteristics of the final material.

## Figures and Tables

**Table 1:** Types of natural fillers and their chemical composition.

Natural Fillers	Chemical Composition of Fillers		
	Cellulose, % by Mass	Lignin, % by Mass	Hemicellulose, % by Mass
Rice husk	28–45	12–16	23–28
Sunflower husk	36	26	25
Wood flour	43	20	26

**Table 2:** Composition recipe.

Filler	Content of Components, wt.-%			
	Polypropylene	Antioxidant	Natural Filler	Modifier
Wood flour	49.9	0.1	50.0	-
	49.9	0.1	49.5	0.5
Rice husk	49.9	0.1	50.0	-
	49.9	0.1	49.5	0.5
Sunflower husk	49.9	0.1	50.0	-
	49.9	0.1	49.5	0.5

**Table 3:** Surface energy characteristics.

Sample	$\gamma_{s}^{ab}$, mH/m	$\gamma_{s}^{d}$, mH/m	$\gamma_{s}=\gamma_{s}^{ab}+\gamma_{s}^{d}$, mH/m
Polypropylene	1.5	27.1	28.6
Wood flour (Control)	12.76	34.71	47.47
Wood flour (Control + Modifier)	8.46	32.67	41.13
Rice husk (Control)	8.82	37.46	46.29
Rice husk (Control + Modifier)	7.9	35.40	43.31
Sunflower husk (Control)	9.45	34.12	43.57
Sunflower husk (Control + Modifier)	8.52	29.23	37.75

## Data Availability

Data are contained within the article.
